# Programme of the Eighth Semi-Annual Session of the Central Texas Medical Association

**Published:** 1897-07

**Authors:** 


### Society Notes.

### Programme of the Eighth Semi-Annual Session of the Central Texas Medical Association.

KOSSE, TEXAS, Jnne 20, 1897.

DEAR DOCTOR:—The eighth semi-annual meeting of the Central Texas Medical Association will be held in Waco, Texas, July 13 and 14, 1897.

The meeting will be called to order promptly at 10 o'clock a. m. Let us hope, doctor, that you will put aside the cares of life for two days and join with us in making this one of the most successful and interesting sessions ever held by our Association. We need your assistance and cordially invite you to be present and participate in the pleasures and add to the interest of the meeting by reading a paper or reporting cases.

The first evening's session will be devoted to reports of cases so that no cases will be left undiscussed.

Fraternally yours,

W. R. BLAILOCK, President.

PAPERS.

1. La Grippe: Paper by P. M. Kuykendall, Moody, and O. I. Halbert, Waco.

Discussion by Douglass Harris, Whitney, and W. B. Carpenter, Mart.

2. Continued Malaria Fever: Paper by J. H. Sears, Waco, and R. B. Whiteside, Durango.

Discussion by H. M. Stewart, West, and W. A. Howard, Waco.

3. Nerve Suturing: Paper by M. D. Knox, Hillsboro.

Discussion by W. A. Wood, Hubbard City, and W. Barton, Salado.

4. Therapeutic Use of Veratrum Viridi: Paper by R. L. Kimmins, Iredell.

Discussion by U. H. Nixon, Killeen, and N. A. Olive, Waco.

5. Hydrotherapy: Paper by Walter Allen, Marlin.

Discussion by H. B. Black, Waco, and J. W. Miller, Hillsboro.

6. Treatment of Shock: Paper by A. C. Scott, Temple.

Discussion by H. W. Cummings, Hearne, and J. P. Naylor, Bell Falls.

7. Surgical Operations in the Nose: Paper by W. F. Cole, Waco.

Discussion by J. R. Ferrell, Waco, and R. B. Sellers, Waco.

8. Acne; Varieties and Treatment: Paper by J. B. Brown, McGregor.

Discussion by R. W. Noble, Temple, and J. B. Shellmire, Dallas.

9. X-Rays and Their Uses in Surgery: Paper by Prof. J. E. Thompson, Galveston.

Discussion by A. M. Curtis, Waco, and F. B. Wilkes, Abbott.

10. Choroiditis: Paper by John O. McReynolds, Dallas.

Discussion by C. Guy Reiley, Waco, and J. M. Woodson, Temple.

11. Peritonitis: Paper by Jarrette D. Law, Salado.

Discussion by J. C. Shaw, Stranger, and John N. Johnson, West.

12. Therapeutic Use of Strychnine: Paper by G. B. Foscue, Waco.

Discussion by J. M. Frazier, Belton, and John R. Gillum, Battle.

13. Perineorrhaphy: Paper by W. R. Blailock, McGregor.

Discussion by John D. McIver, Bosqueville, and A. H. Snead, Waco.

14. Hæmorrhoids—Surgical Treatment: Paper by E. C. Gordon, Lott.

Discussion by S. M. Jenkins, Summers Mill, and W. W. Brown, Groesbeeck.

15. Uric Acid Diathesis: Paper by F. C. McRae, Temple.

Discussion by Daniel Parker, Calvert, and J. C. J. King, Waco.

16. Clinic: J. W. Hale, Waco.

17. A New Club Foot Shoe, with Remarks: Paper by M. M. Edmonson, Dallas.

Voluntary papers.

Individual reports of cases.

Miscellaneous business.

At this meeting amendments to the constitution involving the place of meeting of the Association will come up for final discussion. The amendment provides for making the meetings rotate from town to town throughout the district.

MARVIN L. GRAVES, Secretary,
Waco, Texas.

---

TYLER, TEXAS, June 12, 1897.

DEAR DOCTOR:—The next regular meeting of the East Texas Medical Society will take place at Tyler, July 13th and 14th. Will you kindly contribute an article or report a case at that time? Send title of paper to me before July 10th. Your presence is requested.

E. A. WALDERT, Secretary.

N. B.—Fruit Palace opens July 14th.

